# Supplementary material for: Machine learning reveals cryptic dialects that explain mate choice in a songbird
Source: Nat Commun. 2022 Mar 28;13:1630. doi: 10.1038/s41467-022-28881-w (PMC8960899; doi:10.1038/s41467-022-28881-w)
Supplement: Supplementary file 3 — Description of Additional Supplementary Files [file 41467_2022_28881_MOESM3_ESM.pdf]

Supplementary Movie 1. Association pattern in aviary 1 of populations D_1 and W_1 across 30 days based on pair-wise distance data. Nodes represent individual birds and links represent strong associations. Arrows points to the two individuals that were nearest to a focal bird across an entire day; these are directed relationships, which can be reciprocal or not. Colours represent the cultures (dialects) of individuals: red = D_1, blue = W_1. Shapes represent sex: square = male, circle = female. Cross-fostered individuals are indicated with a star. The dynamic networks were visualized as a movie using the R package ndtv.

Supplementary Movie 2. Association pattern in aviary 2 of populations D_1 and W_1 across 30 days based on pair-wise distance data. Nodes represent individual birds and links represent strong associations. Arrows points to the two individuals that were nearest to a focal bird across an entire day; these are directed relationships, which can be reciprocal or not. Colours represent the cultures (dialects) of individuals: red = D_1, blue = W_1. Shapes represent sex: square = male, circle = female. Cross-fostered individuals are indicated with a star. The dynamic networks were visualized as a movie using the R package ndtv.

Supplementary Movie 3. Association pattern in aviary 1 of populations D_2 and W_2 across 30 days based on pair-wise distance data. Nodes represent individual birds and links represent strong associations. Arrows points to the two individuals that were nearest to a focal bird across an entire day; these are directed relationships, which can be reciprocal or not. Colours represent the cultures (dialects) of individuals: red = D_2, blue = W_2. Shapes represent sex: square = male, circle = female. Cross-fostered individuals are indicated with a star. The dynamic networks were visualized as a movie using the R package ndtv.

Supplementary Movie 4. Association pattern in aviary 2 of populations D_2 and W_2 across 30 days based on pair-wise distance data. Nodes represent individual birds and links represent strong associations. Arrows points to the two individuals that were nearest to a focal bird across an entire day; these are directed relationships, which can be reciprocal or not. Colours represent the cultures (dialects) of individuals: red = D_2, blue = W_2. Shapes represent sex: square = male, circle = female. Cross-fostered individuals are indicated with a star. The dynamic networks were visualized as a movie using the R package ndtv.
