# Supplementary material for: Machine learning reveals cryptic dialects that explain mate choice in a songbird
Source: Nat Commun. 2022 Mar 28;13:1630. doi: 10.1038/s41467-022-28881-w (PMC8960899; doi:10.1038/s41467-022-28881-w)
Supplement: Supplementary file 8 — Reporting Summary [file 41467_2022_28881_MOESM8_ESM.pdf]

nature portfolio

Corresponding author(s): Bart Kempenaers

Last updated by author(s): Jan 27, 2022

Reporting Summary

Nature Portfolio wishes to improve the reproducibility of the work that we publish. This form provides structure for consistency and transparency in reporting. For further information on Nature Portfolio policies, see our Editorial Policies and the Editorial Policy Checklist.

Statistics

For all statistical analyses, confirm that the following items are present in the figure legend, table legend, main text, or Methods section.

n/a	Confirmed	
☐	☒	The exact sample size (*n*) for each experimental group/condition, given as a discrete number and unit of measurement
☐	☒	A statement on whether measurements were taken from distinct samples or whether the same sample was measured repeatedly
☐	☒	The statistical test(s) used AND whether they are one- or two-sided<br>*Only common tests should be described solely by name; describe more complex techniques in the Methods section.*
☐	☒	A description of all covariates tested
☐	☒	A description of any assumptions or corrections, such as tests of normality and adjustment for multiple comparisons
☐	☒	A full description of the statistical parameters including central tendency (e.g. means) or other basic estimates (e.g. regression coefficient) AND variation (e.g. standard deviation) or associated estimates of uncertainty (e.g. confidence intervals)
☐	☒	For null hypothesis testing, the test statistic (e.g. *F*, *t*, *r*) with confidence intervals, effect sizes, degrees of freedom and *P* value noted<br>*Give P values as exact values whenever suitable.*
☒	☐	For Bayesian analysis, information on the choice of priors and Markov chain Monte Carlo settings
☐	☒	For hierarchical and complex designs, identification of the appropriate level for tests and full reporting of outcomes
☐	☒	Estimates of effect sizes (e.g. Cohen's *d*, Pearson's *r*), indicating how they were calculated

Our web collection on statistics for biologists contains articles on many of the points above.

Software and code

Policy information about availability of computer code

Data collection	Code is available from https://osf.io/d59rf/files/
Data analysis	Code is available from https://osf.io/d59rf/files/<br>Software used:<br>Sound Classifier tool in Apple Create ML (https://developer.apple.com/machine-learning/create-ml/; Version 1.0; 16019; Apple Inc. 2019) in an Xcode environment (Version 11.7; 11E801a)<br>Sound Analysis Pro (SAP) version 2011.10427<br>R version 4.0.3 (2020-10-10) -- "Bunny-Wunnies Freak Out" Copyright (C) 2020 The R Foundation for Statistical Computing Platform: x86_64-w64-mingw32/x64 (64-bit)<br>R package ‘lme4’ V1.1-26 Bates D, Maechler M, Bolker B, Walker S. Fitting Linear Mixed-Effects Models Using lme4. Journal of Statistical Software. 2015;67(1):1-48. doi: doi:10.18637/jss.v067.i01.<br>R package 'ndtv' version 0.13.0 - see https://cran.r-project.org/web/packages/ndtv/index.html

For manuscripts utilizing custom algorithms or software that are central to the research but not yet described in published literature, software must be made available to editors and reviewers. We strongly encourage code deposition in a community repository (e.g. GitHub). See the Nature Portfolio guidelines for submitting code & software for further information.

Data

Policy information about availability of data

All manuscripts must include a data availability statement. This statement should provide the following information, where applicable:

- Accession codes, unique identifiers, or web links for publicly available datasets
- A description of any restrictions on data availability
- For clinical datasets or third party data, please ensure that the statement adheres to our policy

Data and code is available from https://osf.io/d59rf/files/

Field-specific reporting

Please select the one below that is the best fit for your research. If you are not sure, read the appropriate sections before making your selection.

☐ Life sciences ☐ Behavioural & social sciences ☒ Ecological, evolutionary & environmental sciences

For a reference copy of the document with all sections, see nature.com/documents/nr-reporting-summary-flat.pdf

Ecological, evolutionary & environmental sciences study design

All studies must disclose on these points even when the disclosure is negative.

Study description

Breeding experiment Generation 1. We created four groups of 36 individuals (9 males and 9 females from both a domesticated and a wild-derived population, two groups within each replicate) and put each group separately in an indoor aviary (5m × 2.0m × 2.5m). All individuals had been reared normally by their genetic parents in similar breeding aviaries, were inexperienced (never mated before) and unfamiliar to all opposite-sex individuals. In replicate 1 (W1 – D1, starting December 2016), birds were 142 ± 32 days old at the start of the experiment (range: 101-191 days); in replicate 2 (W2 – D2, starting March 2017), birds were 241 ± 47 days old (range: 151-306 days). In each aviary, we provided nest material and nest boxes to stimulate breeding and observed pair-bonding behaviour for ca. 60 hours spread over 14 days. Two observers recorded all instances of allopreening, sitting in bodily contact, and visiting a nest box together, which reflects pair bonding64.

In total, we observed 3,166 instances of heterosexual association among the 4 × 36 individuals (Supplementary Table 3). We defined a pair-bond between two opposite-sex individuals if they were recorded in pair-bonding behaviour at least five times (mean: 22 ± 14 SD, range: 5 – 73). This cut-off was chosen (blind to the outcome of data analysis) based on the frequency distribution showing a clear deviation from a random, zero-truncated Poisson distribution (Supplementary Figure 8). Using this definition, we identified a total of 60 pairs (30 in each replicate). Of all females, 48 and 6 had a pair-bond with one and two males, respectively (18 females remained unpaired). Conversely, 34, 10, and 2 males had a pair-bond with one, two, and three females, respectively (26 males remained unpaired).

Cross-fostering for Generation 2 experiments. After the breeding experiment of Generation 1, in 2017, we established two different cultural lineages within each genetic population by cross-fostering eggs, either within or between populations (Fig. 3). For this purpose, we used 16 aviaries (four per population), each containing 8 males and 8 females of the same population (Generation 1). Individuals were allowed to freely form pairs and breed. We reciprocally exchanged eggs shortly after laying between two aviaries per population (within-population cross-fostering) and between pairs of aviaries from different populations (between-population cross-fostering). This resulted in four cultural lineages per replicate (DD, DW, WD, and WW; Fig. 3). Each lineage was maintained in two separate breeding aviaries to ensure the availability of unfamiliar opposite-sex Generation 2 individuals from the same line. Offspring remained with their foster parents until they reached sexual maturity, when the following experiment started.

Social experiment Generation 2. Between December 2017 and March 2018, we put 4 groups of individuals (two groups for each replicate) in indoor aviaries (same as in Generation 1 experiment). Each group consisted of 10 males and 10 females from each of the cross-fostered groups DD, WW, DW and WD, i.e. a total of 80 birds per aviary, except that one aviary of replicate 2 only consisted of 63 individuals (7DD, 8WW, 8DW and 8WD) due to a shortage of birds. In replicate 1 (W1 – D1, starting December 2017), birds were 170 ± 25 days old at the start of the experiment (range: 105-199 days); in replicate 2 (W2 – D2, starting January 2018), birds were 200 ± 29 days old (range: 120-241 days). We recorded the position of individuals using an automated barcode-based tracking system31. We fitted each individual with a unique machine-readable barcode (Supplementary Fig. 4a) and placed eight cameras (8-megapixel Camera Module V2; RS Components Ltd and Allied Electronics Inc.), each connected to a Raspberry Pi (Raspberry Pi 3 Model Bs; Raspberry Pi Foundation) in each aviary. For 30 consecutive days, the cameras recorded individuals at six perches and at two feeders (Supplementary Fig. 4b, c). Between 05:30 and 20:00, when lights were switched on, each camera took a picture every two seconds.

Each day, pictures stored on the Raspberry Pis were downloaded to a central server and processed using customized scripts. The customized software used the PinPoint library in Python65 to identify each barcode in each picture, allowing us to simultaneously track the position and orientation of each individual (Supplementary Fig. 4b) for the duration of the experiment. The tracking system generated 118 million observations across all four aviaries (Supplementary Fig. 4c). From these data, we extracted the average distance between the male and the female (in mm) for each male-female dyad, either daily or across the entire 30-day period (for comparison, such distance data were also extracted for all male-male and all female-female dyads). We used this dataset to identify the nearest opposite-sex individual for each of 151 males and females (55% of these 151 associations were reciprocal). Out of 151 nearest males to females, 74 (49%) paired with that female in the following breeding experiment (see below) and this proportion strongly increased as the average distance between partners decreased (Supplementary Figure 9).

Breeding experiment Generation 2. Immediately after the social experiment, we moved each group into a separate semi-outdoor aviary (5 m × 2.5 m × 2.5 m) and provided nest material and nest boxes. During the next two months, three observers scored heterosexual associations to identify pair bonds as described for 'breeding experiment Generation 1' (ca 300 h per replicate). In total, we observed 6,072 associations involving 284 individuals (Supplementary Table 3). Consistent with the previous experiment, we

defined a pair-bond when a male-female dyad was observed in pair-bonding behaviour at least five times during the entire experiment (mean: 18 ± 13 SD range: 5 - 61; Supplementary Figure 9). Using this definition, we identified 147 pairs (79 pairs in replicate 1 and 68 in replicate 2). Of all males, 97, 22 and 2 had a pair-bond with 1, 2 and 3 females, respectively (27 males remained unpaired). Conversely, 99, 21 and 2 females had a pair-bond with 1, 2 and 3 males (26 females remained unpaired).

Breeding experiment Generation 3. Between April and December 2018, we housed the four cultural lineages (DD, WW, DW and WD) separately again. We placed 8 males and 8 females in each of 16 breeding aviaries (four per lineage) and allowed them to freely form pairs and breed. The offspring belong to four lineages (Fig. 3): two lineages with individuals that were raised by parents that had not been cross-fostered between the domestic and wild-derived population (DDD and WWW) and two lineages with individuals from the same genetic background, but where their parents had been cross-fostered and raised by the other population (DDW and WWD).

Between December 2018 and February 2019, we put four groups of 36 birds (two per replicate, i.e. 2 with 18 DDD and 18 DDW individuals and 2 with 18 WWW and 18 WWD individuals; 9 males and 9 females per lineage; Supplementary Table 3) in an outdoor aviary (same as above). In replicate 1 (W1 – D1, starting December 2018), birds were 172 ± 44 days old at the start of the experiment (range: 131-195 days); in replicate 2 (W2 – D2, starting January 2019), birds were 191 ± 40 days old (range: 122-230 days). During 14 days, two observers recorded all pair-bond behaviours as described under ‘breeding experiment Generation 1’. In total, we observed 3,378 instances of pair-bond behaviour involving 137 individuals (Supplementary Table 3). As above, we defined a pair-bond when a male-female dyad was observed in pair-bonding behaviour at least five times during the entire experiment (mean: 18 ± 11 SD, range: 5 - 47; Supplementary Figure 9). We identified 82 pair bonds (37 in replicate 1 and 45 in replicate 2). Of all males, 34, 16, 4 and 1 had a pair-bond with 1, 2, 3 and 4 females (17 males remained unpaired), respectively. Conversely, 42, 16, 1 and 1 females had a pair-bond with 1, 2, 3 and 5 males (12 females remained unpaired).

Morphological measurements. After birds had reached sexual maturity (> 100 days of age), we measured body mass (to the nearest 0.1g), tarsus length (to the nearest 0.1mm), and wing length (to the nearest 0.5mm) of all individuals (all measured by WF). We included these three variables in a principal component analysis (PCA) and used the first principal component (PC1, 67% of variation explained) as a measure of body size.

Song recording and analysis approach. We recorded the songs of the parental males from Generation 1 (16 aviaries x 8 males = 128 males, of which 122 were successfully recorded between November and December in 2017) and of their offspring (Generation 2; 146 out of 152 males were successfully recorded between March and May 2018). To elicit courtship song, each male was placed together with an unfamiliar female in a metal wire cage (50 cm × 30 cm × 40 cm) equipped with three perches and containing food and water. The cage was placed within one of two identical sound-attenuated chambers. We mounted a Behringer condenser microphone (TC20, Earthworks, USA) at a 45◦ angle between the ceiling and the side wall of the chamber, such that the distance to each perch was approximately 35 cm. The microphone was connected to a PR8E amplifier (SM Pro Audio, Melbourne, Australia) from which we recorded directly through a M-Audio Delta 44 sound card (AVID Technology GmbH, Hallbergmoos, Germany) onto the hard drive of a computer.

Previous studies that quantified differentiation of songs between zebra finch populations using specific song parameters (e.g. duration and frequency measures) largely failed to detect prominent differences12,49,50. We therefore used the following two approaches (Sound Analysis Pro and Machine Learning) in order to quantify the extent to which a given male’s song resembled the songs of other males.

Song similarity analysis with SAP. Using Sound Analysis Pro (SAP) version 2011.10427 we quantified song similarity (ranging from 0 to 100) by direct pairwise comparison of song motifs (the main part of a male’s song that is stereotypically repeated and about 0.8 sec long, excluding introductory syllables). Pairwise comparisons of two males (based on one representative motif recording per male) revealed higher within-population similarity than between-population similarity (Supplementary Table 2, data from Generation 1). Further, for offspring that were cross-fostered between populations (N = 73 males from Generation 2) song similarity to their foster father was higher than song similarity to their genetic father (80 versus 68, paired t-test: $p < 0.0001$). For each of the 146 recorded males of Generation 2, we calculated three measures of song similarity with regard to each of the females encountered in the social experiment with automated tracking of birds. (1) ‘SAP song similarity to foster father’: the pairwise similarity between the motif of the focal male and the motif of the foster father of the focal female. (2) ‘SAP song similarity to parents’: we first combined the song motifs of all 8 parental males that were present in the female’s rearing aviary (Generation 1) into a single ‘super-motif’ (simply placing all recordings into a single sound file) and then calculated the similarity of the motif of the focal male to this super-motif from the female’s rearing aviary. (3) ‘SAP song similarity to peers’: we combined the song motifs of all 7-10 recorded peer males present in the female’s rearing aviary (Generation 2) into a single ‘super-motif’ and calculated the similarity of the motif of a focal male to this super-motif.

Song categorization based on machine learning. We used the Sound Classifier tool in Apple Create ML (https://developer.apple.com/machine-learning/create-ml/; Version 1.0; 16019; Apple Inc. 2019) in an Xcode environment (Version 11.7; 11E801a) to (1) assess the proportion of individual song recordings that can be correctly assigned to their population (Table 1), and (2) to quantify the confidence with which songs of individual males are assigned to a given population (Fig. 7). We interpret the former as a measure of overall divergence between two populations and the latter as a measure of song similarity of an individual to a population. As input we used two recordings for each individual male (mean ± SD duration per recording: 6.8 ± 1.6 sec, range 4.5 – 10.2 sec; n = 536).

To quantify the overall classification success, we first trained the sound classifier on two categories of songs (e.g. songs of population W1 versus D1) using all available recordings from individuals from Generation 1 (i.e. 30-32 males per population, represented by 60-64 song recordings). After the training phase, the software reports a validation statistic, which is the proportion of training songs that are classified correctly with the algorithms derived from the training set (this value has to be interpreted cautiously, see below). For independent validation, we then tested the classification success (proportion of tested songs that are classified correctly) on recordings from individuals from Generation 2 (i.e. 17-20 males per population, using 34-40 songs). We did this separately for the males that had been cross-fostered within and between populations. All steps (training, validation, and testing) were carried out for all six pairwise combinations of the four captive populations used in this study.

Besides reporting a classification result for each tested recording, the sound classifier also reports a confidence statistic (complementary likelihoods of belonging to each of the two classes) for each 1 sec interval of the recording in a sliding window with 50% overlap. As the classification success and overall confidence may increase with the length of recording, we trimmed all recordings to 4.5 sec and averaged for each recording the confidence scores for a given class from the first (0 to 1 sec) to the last (3.5 to 4.5 sec) time interval. We interpret this mean confidence value in belonging to a certain class as a measure of similarity to that class. In analogy to the similarity values from SAP (see above) we retrieved ‘ML similarity values’ from the perspective of each female from Generation 2 with regard to the males from her rearing aviary. Hence, we trained the sound classifier to distinguish the songs of the 8 parental males (Generation 1) of a female’s rearing aviary from those of the other population type which the female would later encounter (e.g. W1 vs D1, 16 parental recordings each). The classifier was then tested with each of the songs of the (usually 40) males that the female would later encounter, to obtain values of their song similarity to the parents in her rearing aviary (‘ML song similarity to parents’). The similarity values from each of the two recordings of a male were averaged (repeatability: r = 0.88, n = 584

pairs of values from 146 males, each combined with four female rearing aviaries). Similarly, we trained the sound classifier using the respective peer males of Generation 2 (males with whom females grew up in their rearing aviary) in contrast to peers from the other population type, to obtain values of similarity of males to those peer members ('ML song similarity to peers', repeatability r = 0.91, n = 584).

To further validate the classification procedure we ran a negative control by training on two sets of 25 songs (mean duration 16.4 sec per recording) from a single population. Classification success was 49.5% in the testing phase, which is close to the 50% chance level. Note that validation after training indicated a 80% classification ability within the training set, indicating that the utility of a trained classifier should be judged by independent testing and not from the validation percentages. We recorded all birds in one of two identical sound-proof chambers (see above), which ensured that classification success during testing stemmed from properties of the recorded songs rather than from idiosyncratic background noises. For example, such background noises might differ when wild populations would be recorded in their respective natural habitats. To avoid this problem, both sound-proof chambers were used about equally often in each of the four populations within each of the two recorded generations. As the two generations were recorded about one year apart, confounding effects of background noise that would closely match the offspring to their population of foster parents (depending on whether they had been cross-fostered between or within populations) can be excluded. No pre-processing of sound files (e.g. noise-reduction) was carried out prior to analysis.

Data analysis. To investigate whether pair-bonding and heterosexual social associations depended on culture (population of rearing) or on genetic background (population of origin), we used two statistical approaches. First, for the data set of identified pairs, we tested whether the observed degree of mating assortment by either population of rearing or by population of origin differed from expectations under random mating (50:50), using an exact binomial test. We tested each replicate separately for each of the three generations.

Second, for the data set on heterosexual interactions (also including individuals that were defined as unpaired, see above), we constructed a social network, where nodes represented individuals and edges represented pair-bonding interactions between individuals. We did this separately for each aviary and for each breeding experiment (Generations 1-3). We then quantified the extent to which social interactions were clustered by culture by calculating the assortativity coefficient for each social network66. The assortativity coefficient is a network version of the Pearson's correlation coefficient, where the value from -1 to 1 reflects the tendency for individuals with similar attributes (here: population of rearing) to be associated in the network (r=1), randomly associated (r=0), or disassociated (r=-1). We used permutation tests to assess whether the association by culture was significantly non-random44. To obtain a p-value, we randomly re-allocated the phenotype value (population of rearing) across the nodes in the network (10,000 times) and calculated the assortativity coefficient for each permutated network. The p-value then equals the proportion of assortativity coefficients that were larger than the observed coefficient.

For the 'social experiment generation 2', we derived a daily social network using the pair-wise distance data and compiled this into a dynamic network video across the 30 days to visualise the association pattern. We also calculated the corresponding assortativity coefficients by culture for each day. Further, we analysed these daily social networks across 30 days within and between sexes to reveal the temporal patterns of assortment by song or by population of origin (genetic background) of each sex. This is for investigating the differences of social patterns between heterosexual relationships and same-sex relationships.

We tested whether the daily pair-wise distance (from the social experiment Generation 2) can be explained by cultural (song) similarity and by genetic (size) similarity between females and males that participated in this social experiment. We used generalized mixed-effect models67 with distance of each male-female combination as the response variable and with female identity (151 levels), male identity (151 levels), the combination of male and female identity (pair ID: 5,752 levels), and the combination of the male's and the female's rearing aviaries (64 levels) as random effects. As fixed effects of interest, we fitted several categorical predictors that distinguish different types of male-female combinations (for details see Supplementary Table 5) and several continuous predictors (measures of body size and song similarity, see above) that reflect individual-specific traits in a male-female combination.

Research sample	Study populations. We used four captive zebra finch (Taeniopygia guttata) populations that are genetically differentiated due to founder effects and selection (see Supplementary Fig.1 & Fig. 2): two domesticated populations (D1 and D2) that have been maintained in captivity in Europe for about 150 years and two populations (W1 and W2) that have been taken from the wild about 10-30 years ago (see Supplementary Fig. 1). We ran all experiments in two independent replicates. We used individuals from populations D1 and W1 for replicate 1 and individuals from D2 and W2 for replicate 2
Sampling strategy	Sample sizes were maximised within given constraints (availability of 4 populations, 16 aviaries for breeding, annual breeding success for maximal statistical power). The sampling procedures are described above.
Data collection	Data collection was carried out by DW, KM, and YP using the methods described above. Sampling effort followed previously established routines and was never contingent on preliminary data analysis.
Timing and spatial scale	Data collection periods are described above. Sampling effort followed previously established routines and was never contingent on preliminary data analysis.
Data exclusions	No data were excluded.
Reproducibility	The experiment was carried out in two independent replicates (populations D1 and W1 versus populations D2 vs W2) to allow assessing the replicability of our findings. All attempts of replication are reported.
Randomization	Birds within their populations were randomly assigned to treatment aviaries
Blinding	In all reported experiments, the treatment of birds (cross-fostering history) was unknown to observers, but the concealment of population differences in body size was not possible.

Did the study involve field work? ☐ Yes ☒ No

Reporting for specific materials, systems and methods

We require information from authors about some types of materials, experimental systems and methods used in many studies. Here, indicate whether each material, system or method listed is relevant to your study. If you are not sure if a list item applies to your research, read the appropriate section before selecting a response.

Materials & experimental systems

n/a	Involved in the study
x	Antibodies
x	Eukaryotic cell lines
x	Palaeontology and archaeology
	x Animals and other organisms
x	Human research participants
x	Clinical data
x	Dual use research of concern

Methods

n/a	Involved in the study
x	ChIP-seq
x	Flow cytometry
x	MRI-based neuroimaging

Animals and other organisms

Policy information about studies involving animals; ARRIVE guidelines recommended for reporting animal research

Laboratory animals	Taeniopygia guttata castanotis, 591 individuals, about 50% female, age at start of observation of pairing behaviour 185 days (range 101-304).
Wild animals	No wild animals were used in the study
Field-collected samples	No field-collected samples were used in the study
Ethics oversight	This study was carried out within the frame of our housing and breeding permit (311.4-si) granted by the Landratsamt Starnberg, Germany. Attachment of backpacks was approved by the Regierung von Oberbayern, Germany (ROB-55-2-2532. Vet_02-17-211).

Note that full information on the approval of the study protocol must also be provided in the manuscript.
